# Behavior change beyond intervention: an activity-theoretical perspective on human-centered design of personal health technology

**DOI:** 10.3389/fdgth.2026.1754399

**Published:** 2026-04-20

**Authors:** Victor Kaptelinin, Kaan Kilic, Helena Lindgren

**Affiliations:** 1 Department of Informatics, Umeå University, Umeå, Sweden; 2 Department of Computing Science, Umeå University, Umeå, Sweden

**Keywords:** activity theory, behavior change, development, intelligent digital companions, intervention, persuasive technology

## Abstract

**Introduction:**

Modern personal technologies, such as smartphone apps with artificial intelligence (AI) capabilities, have a significant potential for helping people make necessary changes in their behavior (e.g., adopt healthier lifestyles). Current research highlights that realizing this potential through the design and use of personal technologies calls for a critical reappraisal of the role of healthcare interventions as the driving force of behavior change and requires a more explicit focus on human agency and experience.

**Methods:**

This paper contributes to this line of investigation by developing and presenting a conceptual framework, informed by activity theory, which views behavior change as an outcome of the combined agencies of healthcare professionals, technology designers, and, most importantly, the persons themselves.

**Results:**

According to the framework, the process of behavior change can be represented as a transformation, achieved through an interplay between the activity systems of intervention, development, and empowerment. In addition to presenting the conceptual framework, we offer insights into how these ideas can be implemented through examples of digital companions.

**Discussion:**

Implications of the analysis for the design of personal technologies for supporting healthier lifestyles, with a special focus on intelligent digital companions, are discussed.

## 1 Introduction

### 1.1 Background

Making positive lifestyle changes, such as quitting smoking, exercising more, improving eating habits, and more effectively managing stress, is a major factor preventing many health problems and increasing the quality of life overall (1, 2). However, people who can significantly benefit from changing their behavior may find it difficult to do that (3). A common way to help people achieve the needed change is using mobile, personally owned technologies, most notably smartphones (4). Mobile technologies are currently widespread and increasingly powerful and versatile, due to ever more advanced processors, sensors, computing capabilities, communication features, and the growing number of available apps. Their ubiquity and rich functionality make mobile technologies a potentially effective tool for supporting people in their transition to healthier behavior, e.g., by providing reminders, motivation, and advice (5).

In the last decade, significant efforts have been made to create mobile apps for behavior change due to the outreach mobile apps have to target groups (6). Villalobos-Zúñigaa and Cherubini (7) assess the number of such apps as exceeding 100,000. Though some health and well-being apps take behavior theories, principles, or strategies into consideration in their development, many often do not include theoretical constructs (8). Accordingly, the effectiveness (or even safety) of such apps is often questionable (9). Another problem with existing mobile apps for improving health is that they are often designed without properly understanding the intended users and the contexts of use (7, 10). As argued by Hicks et al. (11), to fully realize the potential of mobile technologies for public health promotion, it is crucial that the technologies be designed from a human-centered approach, which starts with understanding intended users and their use contexts. However, while human-centered design has become the dominant perspective in Human-Computer Interaction (HCI) and interaction design (12), so far, the perspective has made a limited impact on the practice of designing mobile apps for eHealth. In particular, in the design of mobile apps for stress management, HCI methods and concepts were found to be used mostly to ensure usability and engagement (13).

This paper contributes to existing efforts to achieve a more systematic and advanced application of human-centered design in personal health technology, moving beyond usability and engagement by proposing a conceptual framework for human-centered design, informed by activity theory (14–16). The framework views technologies for behavior change as embedded in real-life, dynamic social contexts, and aims to provide a balanced account of both healthcare intervention and persons' own agency. A central feature of the framework is differentiating between two orientations of behavior change: *exogenous* change which is driven by external agents such as healthcare professionals, and *endogenous* change which is driven by individuals themselves. These terms emphasize distinct placements of agency and aid in analyzing how technologies can support different aspects of behavior transformation.

### 1.2 Methodological approach

The paper presents a conceptual analysis integrating theoretical insights from behavior change research and human–computer interaction (HCI). The objective of the analysis is to develop a design-oriented framework for behavior change, making it possible to realize the full potential of modern personal, and especially intelligent, technologies. The analysis proceeded in three stages.

We started by reviewing the theoretical orientations in digital health and HCI research, which have been particularly influential in current work in technology-supported behavior change. The following orientations were identified and reviewed:

– intervention-oriented models, which provide operationalizable constructs for digital health interventions (e.g., (17, 18),
– autonomy-oriented perspectives, most notably those informed by self-determination theory (19), which emphasize intrinsic motivation and the internalization of behavior regulation (e.g., (20, 21), and
– experience- and activity-oriented perspectives, which highlight lived experience, meaning making, and development [e.g., (22–24, 53)].

The analysis revealed a recurring distinction between behavior change driven primarily by external actors, e.g., healthcare professionals and behavior change driven by persons themselves. It was concluded that there is a need for a design-oriented framework that more fully acknowledges the interplay between external actors' agency and persons' own agency.

Activity theory (14, 15) was selected as a primary theoretical influence for the development of such a framework. The theory understands human activity as socially mediated, culturally developed, and situated in networks of interacting activity systems (14, 15), which makes it particularly suitable for the design of technological support for behavior change as supporting an interplay of individual human agencies and the social context.

The design orientation of the analysis and its focus on both external actors' agency and persons' own agency when applying Engeström's network of activity systems model resulted in identifying three interacting components of the model: (a) an intervention activity system, representing the activities of healthcare professionals and institutions (exogenous intent); (b) a development activity system, representing the individual's own efforts to transform everyday practices and integrate healthier behaviors into their life context (endogenous intent); and (c) a design/empowerment activity system, representing the activities of technology designers who create artifacts intended to support both intervention and personal development (augmentative intent). Conceptualizing behavior change as an interaction between these systems makes it possible to analyze how different actors, tools, and goals interact during the process of behavior transformation.

By examining how the interaction between the three activity systems generates recurring design challenges for personal health technologies, we derived five design concepts: interaction spaces, contradiction-driven development, holistic transformation, appropriation, and transitions in agency. Each design concept reflects a particular implication of the analysis for technology design.

To demonstrate how the conceptual framework can be interpreted in relation to existing technologies and emerging design directions in digital health, we provide a set of examples: the StarCoach system, diabetes management applications, and conversational AI companions. These examples were chosen because they represent different design orientations, including clinician-driven systems, user-driven reflective technologies, and hybrid systems, and therefore help illustrate the analytical distinctions proposed in the framework.

The resulting framework should therefore be understood as a conceptual design framework intended to support analysis and design of behavior change technologies rather than as a tested intervention model. Its purpose is to clarify how different forms of agency interact in technology-supported behavior change and to provide a theoretically grounded basis for developing human-centered digital health systems.

## 2 Technology-supported behavior change: an overview of related work

Healthcare assesses illness, the causes of illness, and intervenes to cure illnesses. Interventions that are based on evidence or best practice are recommended and prescribed. The treatment period is defined, and whether the intervention has the intended effect is followed up, partly to judge whether the intervention needs to be adjusted or replaced with a different one. However, non-communicable diseases such as cardio-vascular diseases and mental health conditions that are increasing substantially in society, are developed over time and are to a notable extent dependent on lifestyle behavior and environmental factors. Interventions such as systematic health checkups in combination with motivational interviewing by a trained nurse to elicit health behaviors and what needs to be changed have shown to cause a decrease in cardio-vascular disease in a population (25). Another example of addressing the need for health behavior change is the praxis of prescribing patients “physical activity on receipt”.

There are numerous examples of using digital technology as a tool to promote health behavior (4). Most target one particular health behavior to be changed, such as sedentary lifestyle, tobacco or alcohol use, or unhealthy food habits. 92% of reviewed studies report evidence of effect (4). However, many such uses are not evaluated with a diverse and sufficiently large population over time, and many still are not guided by the theories on behavior change, e.g., (26). Further, digital technology to change health behavior is researched in different research communities with limited interaction, most notably, the behavioral sciences and technological communities (27). Since these communities provide complementary perspectives on technology for health behavior, the evolvement of the two perspectives is briefly introduced below.

### 2.1 Persuasive technology

Interactive information technology designed for changing users' attitudes or behavior is known as persuasive technology (28). Fogg provided a definition of persuasion as “*an attempt to shape, reinforce, or change behaviors, feelings, or thoughts about an issue, object, or action*”. The definition implies that there has to be an intent, mediated through persuasive technology, which Fogg describes as being one of three types: *endogenous*—in his definition stemming from the intent of the designer or organization to embed persuasiveness into the software; *exogenous*—the environment; or *autogenous*—stemming from the individual themselves. He suggested to the HCI community that focus of research should be on the first kind, which has also been the case, in particular, in the domains of recommender systems (29) and behavior change for improving health, exemplified by the works of Michie and others (18). The fields of persuasive technology and recommender systems are rapidly advancing through artificial intelligence (AI) technology development, visible by the numerous recommender systems and social bots distributed through social media.

Fogg (28) also emphasized the importance of persuasive systems' *ethical aspects*. Oinas-Kukkonen & Harjumaa (30) embedded ethical aspects in their definition of persuasive systems: “*computerized software or information systems designed to reinforce, change or shape attitudes or behaviors or both without using coercion or deception*”. They also developed a framework for Persuasive System Design (PSD) with seven postulates embedding ethical principles, and 28 design principles sorted into primary task support, dialogue support, system credibility support, and social support. The proposed definition is challenged by Kampik et al. (31) and Tengland (32) also elicited the ethical challenges when behavior change is the aim and provided motivations for a shift of aim towards empowerment. More recently, the increased use and awareness of AI technology among researchers and in society are increasing the focus on ethical aspects and consequences of using AI technology (33), such as recommender systems, and dialogue systems based on large language models and generative AI.

Fogg (17) presented a behavior model for design with a particular focus on behavior (the Fogg Behavior Model, FBM). Behavior is defined as the product of three factors: Motivation, Capability, and Triggers, as well as their sub-components. The core motivators in the model relate to positive and negative emotions, and triggers and capabilities relate to barriers and facilitators for enacting the target behavior.

### 2.2 Behavior change mechanisms

Michie and colleagues in the domain of behavioral sciences formed a model similar to Fogg's, the “COM-B system” as a model of the individual's sources of behavior targeted for behavior change interventions (18). As the Fogg's model, the COM-B model can be seen as a meta-model of the different theories on behavior change. The COM-B model consists of the conditions Capability (psychological, physical), Opportunity (social, physical), and Motivation (automatic, reflective). The COM-B system of conditions forms the hub of a wheel of behavior change.

They also identified and embedded in the wheel a layer of nine *intervention functions* aimed at addressing *deficits* in one or more of the conditions. The nine intervention functions are restrictions, environmental restructuring, education, persuasion, incentivization, coercion, training, enablement, and modeling. Their work was extended into the taxonomy and, more recently, ontology of behavior change techniques (BCT) (34, 35).^1^ The BCTs connections to mechanisms of action (MoAs) such as motivation or self-efficacy (and other fundamental components of theories on behavior change) are explored by experts in behavior change (36, 37). The model and mechanisms are evaluated, or designs and results are presented using the models in studies of implemented behavior change interventions.

### 2.3 Intelligent digital companions

Numerous research communities (e.g., computer science, psychology, etc.) conjointly are exploring how to design behavior change technologies and examining how specific BCTs might influence individuals' MoAs. They measure this by tracking changes in attitudes and/or behavior change that result from digital interventions. Most of these interventions do not embed characters in the form of a digital companion or coach as a counterpart in the interaction. However, this is expected to change with the new technologies for dialogs using generative AI and large language models. Effects of designing intelligent digital companions as implementation of BCTs have been studied reporting positive results on motivation and self-efficacy (38–40).

Adam et al. (41) reviewed various aspects of shaping a digital companion as a human, distinguishing between avatars that the human controls, and digital embodied agents that exhibit system-generated behavior. In interventions where digital companions were designed to enact one or more social roles, the roles of a coach or a counselor were the most common.

### 2.4 Behavior change as intervention

As discussed above, in healthcare practice, behavior change activities are commonly denoted as “interventions”. The term implies that health professionals or other “external” entities, experts or authorities^2^, are responsible for making qualified decisions about a treatment and following up on its effects. The persons^3^ themselves are typically regarded as “patients” and “participants” who agree to, and are expected to adhere to and to implement, the decisions (e.g., “self-administer” an intervention).

In many traditional medical fields, such as surgery, interventions are characterized by a direct impact of medical experts on the object of intervention (e.g., the patient's body). The patients usually do not play an active role in the process and can even be deliberately prevented from interfering, e.g., by being subjected to anesthesia. In addition, such medical interventions are typically limited in time and take place in the settings controlled by health professionals (e.g., medical facilities).

While employing behavior change techniques by health professionals falls into the general category of “interventions”, the meaning of the term in this context is, arguably, somewhat different. In case of behavior change, interventions in the proper sense play a more limited role and do not represent the whole set of activities involved in a behavior change. Health professionals' efforts may be crucial for initiating, planning, and assessing the process of behavior transformation. However, the transformation as such is inevitably performed by the person themselves, and person's own decisions regarding how, or even if, a change is to be implemented.

### 2.5 Behavior change beyond intervention: supporting persons' agency and autonomy

In human-technology interaction research, there has been a long-standing tradition of studies of human agency and autonomy (42). In line with this tradition, a range of conceptual analyses, empirical studies, and design explorations in the field, especially in recent years, have been focusing on users of behavior change technologies as subjects transforming their own behavior. The research identifies the limitations of understanding behavior change as an external intervention and highlights the need to understand the users' experiences, meaning making, and life transformation in general. The studies have been conducted from a variety of theoretical standpoints (43), including self-determination theory, phenomenology, and activity theory.

An especially influential perspective has been *self-determination theory* (SDT (19),), which differentiates between intrinsic and extrinsic motivation and considers autonomy an innate, essential psychological need (along with competence and relatedness). According to SDT, a key condition for sustainable behavior change is the development of an internal perceived locus of causality, which corresponds to either intrinsic motivation or internalized (integrated) extrinsic motivation.

Villalobos-Zúñiga and Cherubini (7) offer an SDT-based taxonomy of design features of behavior change apps. Eilert et al. (44) argue that maintaining physical activity as part of osteoarthritis therapy requires long-term development of intrinsic motivation and self-regulation skills. Gerstenberg et al. (45) propose the technology-mediated regulatory transformation (TMRT) framework for the design of technological support for behavior change. The framework is based on SDT's notion of internalizing extrinsic motivation along the continuum of external, introjected, identified, and integrated regulation (19). According to TMRT, the use of technology can facilitate the internalization of extrinsic motivation e.g., by “designing for friction”, and include the use of tangible interactive artifacts, such as *Equi (*20). A study of user experience of Digital Diabetes Management (DDM) technologies revealed that the experience is significantly affected by the persons' need for autonomy. To fulfill this need, people may use the system in ways, potentially deviating from medical recommendations (21).

A *phenomenological* perspective, emphasizing the centrality of human lived experience (46, 47), has been adopted by Rapp et al. (22) and Rapp and Boldi (23). It is argued that behavior change should be understood as a meaningful transformation of a person and their circumstances, which may take place over several interconnected domains. Rapp et al. (22) report empirical evidence that demonstrates the fundamental importance of internal aspects of change, such as subjective assessments or alignment with one's values. Rapp and Boldi (23) propose an existential model of behavior change, show that people experience the change as deeply tied to personal and existential meanings, and call for future-oriented design strategies.

Several studies have been informed by *activity theory,* an interdisciplinary approach foregrounding purposeful, social, mediated human activity as a generative force behind human development (14, 16, 48). Almalki et al. (49) proposes a framework for self-quantification (SQ), employing six activity theory-based constructs: users, tracking tools, health objectives, division of work, community or group setting, and SQ plan and rules. A study of the management of chronic heart conditions (50) revealed the centrality of both patients' self-reliance and patient-clinician cooperation in achieving successful management.

The use of activity theory was found especially helpful in behavior change research specifically dealing with sustainability. Li and Landay (51) propose an activity theoretical framework for prototyping applications supporting habit formation. Selvefors et al. (24) found that while having an explicit goal to conserve energy, the participants often experienced conflicts with other competing goals, which could make energy conservation challenging. Svensson (52) analyzed the effect of contradictive motives of healthcare professionals on the sustainability of eHealth interventions. Chu et al. (53) proposed a design toolkit for integrating sustainable design goals into users' dynamic activities. Chu et al. (54) present a meta-synthesis of the uses of activity theory in analysis and design of sustainable behavior, highlighting that the theory considers sustainability as a system property embedded in the complex socio-technical context of people's everyday life.

Finally, Ayobi et al. (55) report on the design and assessment of an app supporting personally meaningful self-tracking in multiple sclerosis self-care. Ayobi et al. (56) analyze a co-designing of a diabetes self-management application to identify opportunities for supporting self-care agency.

### 2.6 The need for further development of conceptual account of agency and autonomy in behavior change

The discussion in the previous section suggests that there are two general theoretical orientations in current research on technological support for people as subjects changing their own behavior. On the one hand, SDT-based research emphasizes the “external-internal” dimension of behavior regulation and different types of motivation. Accordingly, the role of technology is considered to be a facilitator of a transition to *autonomy*, moving from externally controlled to internally regulated behavior. For instance, TMRT (45) offers concepts and design solutions for supporting the user's progress from external to introjected to identified to integrated regulation.

On the other hand, both phenomenology and activity theory, even if from somewhat different perspectives (16),, highlight the importance of understanding the overall personal meaning of behavior change activities in particular life contexts. Accordingly, the aim of technological support is viewed as supporting human *agency* in a broad sense, as a capacity to act to achieve meaningful goals. Arguably, a high level of agency in this sense may involve acting on external rewards if they enable achieving meaningful, high-value outcomes. According to Bennett et al. (42), while the notions of autonomy and agency are often not clearly differentiated in HCI research, their use may reflect important conceptual distinctions characterizing the research. One such distinction, “*independence* vs. *interdependence*”, can be mapped to theoretical orientations in behavior change research, discussed above. SDT-informed studies typically focus on “external-internal” regulation, thus foregrounding one's independence from external pressure, while phenomenology and activity theory by highlighting the embeddedness of behavior change in the context of one's activities, lived experience, and social interactions, underscore the centrality of interdependedness.

Each of these theoretical orientations has its own strengths and limitations. While the focus on interdependence and lived experience expands the scope of analysis, with some notable exceptions, the research within this orientation tends to provide limited clarification of how its conceptual insights, however compelling, can be translated into specific design solutions. SDT-informed analyses, foregrounding autonomy and independence, such the TMRT framework (45), may offer detailed design concepts and solutions but, as observed by Güldenpfennig et al. (57), a strong focus on independence may result in overlooking some directions for supporting behavior change.

We argue that further development of conceptual foundations for technology-supported behavior change requires an elaboration of the “interdependence perspective” into a more specific set of concepts and models. This paper aims to take a step in this direction by introducing an activity-theoretical framework, employing the notion of a “network of activity systems” (15).

## 3 An activity-theoretical framework of behavior change

In this section, we introduce a conceptual framework of technology-supported behavior change as an *interplay* between behavior change driven by external agents such as healthcare professionals (*exogenous* intent*)* and by persons themselves (*endogenous* intent*),* both empowered by technology design (*augmentative* intent). The framework is informed by activity theory (14, 15). To make the link between the framework and the theory explicit, we first present an outline of activity theory's basic concepts and principles. After that, we discuss similarities and differences between activity theory and other perspectives on behavior change, present a framework of behavior change as a network of activity systems, and discuss the implications of the framework for designing technological support for behavior change.

### 3.1 Activity theory: an overview of key ideas

Activity theory is a multidisciplinary conceptual framework (14, 15), which is based on the fundamental notion of “activity” as a purposeful, developing, and mediated interaction between humans (“subjects”) and the world (“objects”). The theory has been employed in a wide range of disciplines and fields of study, including education, organizational learning, and human-technology interaction (16, 58). Activity theory was originally developed by Alexey Leont'ev (14), building on Lev Vygotsky's cultural-historical psychology (59). In turn, Leont'ev's theory served as a basis for a more recent activity-theoretical approach, proposed by Yrjö Engeström (15). Main ideas of each of these approaches are briefly summarized below.

According to cultural-historical psychology, culturally developed tools, through internalization, mediate human mental functions, which dramatically increases the possibilities for humans to control their own behavior (59). Vygotsky's “general law of psychological development” states that each mental function first emerges as an “inter-psychological” one, distributed between the person and other people, and then becomes “intra-psychological”, which means that the person becomes capable of performing the function independently.

Vygotsky's notions of mediation and internalization were employed by Leont'ev's activity theory (14) which provides an account of the human mind as embedded in the overall context of human interaction with the world. Activity, a “subject-object” interaction connecting the human subject and the objective world, is considered a generative force, transforming both the subject and the object. The basic principles of Leont'ev's approach (16, 60) include:

 *object-orientedness:* human activities are always directed at something objectively existing in the world, which gives the meaning to the activities; *hierarchical structure of activity:* “subject-object” interaction is taking place at the levels of activities, actions, and operations, which generally correspond to the questions of *Why?, What?, and How?; an* action can *be poly-motivated*, that is, performed to meet several motives or higher-level actions at the same time; *mediation:* “subject-object” interaction is mediated by culturally developed means, which make it possible for the person to capitalize on previous experiences of other people; e*xternalization/internalization:* there is a dynamic re-distribution of external (physical and/or social) and internal (mental and/or individual) components of activities; and *development:* human activities as undergoing various developmental transformations.

**Figure 1:**
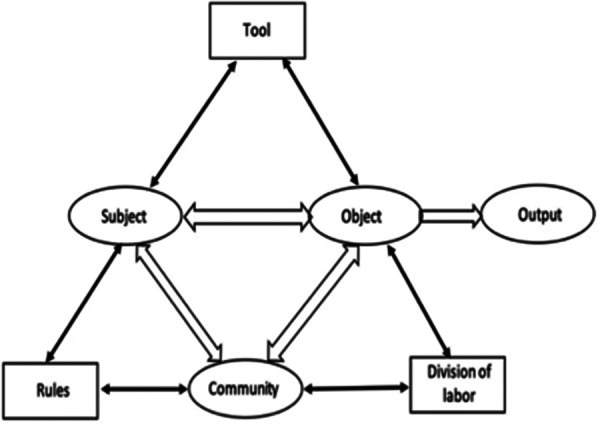
Engeström's activity system model [adapted from (15)] represents human activity as a network of interacting components.

An influential activity-theoretical approach was proposed by Engeström (15, 48), who extended the Leont'ev's notion of activity as “subject-object” interaction by adding “community” as a third component. The extension forms the basis of Engeström's activity system model, shown in Figure 1. The model comprises a three-way interaction between “Subject”, “Object”, and “Community” and three types of mediational means, *Tool*, *Rules*, and *Division of labor*, mediating, respectively, “Subject-Object”, “Subject-Community”, and “Community-Object” interactions. In addition, the model includes “Output”, as the result produced by the activity as a whole.

A major focus of Engeström's framework is on developmental transformations of activity systems. The transformations, according to Engeström, are driven by various types of emerging contradictions in an activity system. In relation to behavior change, the emphasis on contradictions makes it imperative to move beyond designing for isolated actions towards supporting individuals as they begin to view the conflicts and work on resolving them across time. Another fundamental insight of Engeström's approach is that in real-life contexts, one encounters *networks of activity systems*, in which an outcome of an individual activity system may affect a certain component of another activity system or need to be combined with outcomes of other activity systems.

### 3.2 Activity theory vs. other approaches to autonomy and agency in behavior change

Activity theory shares certain similarities with SDT and phenomenology. In particular, like these approaches, activity theory considers humans as subjects, who are ultimately responsible for transforming their behavior. There are also significant differences.

While activity theory and SDT use similar terminology, most notably internalization and contradictions/ tensions [e.g., (20)], the meaning of the terms within these approaches are not the same. Internalization in activity theory refers to the transformation of cognitive and semiotic mental functions rather than motivation. Internalization of motivation, as it is understood in SDT's, roughly corresponds in activity theory to “the shift of the motive to the goal”, when a former goal (an intermediate step for achieving something more important) becomes itself a reason for action. In addition, in activity theory, internalization is complemented by externalization in the process of a continuous re-distribution of activities along the dimensions of “individual/ social” and “physical/mental”. Contradictions as the drivers of development are understood in activity theory in a broader, dialectical sense, as unavoidable tensions within and between the components of an activity system, as well as between different activity systems.

Like phenomenology, activity theory considers human beings and their development as inseparable from the world in which they exist. While sharing a number of basic principles, phenomenology and activity theory are different in their predominant concerns being about, respectively, lived experience and objective structure of the “subject-object” relationship (16).

Regarding the “agency vs. autonomy” distinction (42), the main focus in activity theory is on the subject's agency, that is, the capacity of producing purposeful actions (including both decisional and executional aspects). In certain contexts, and at certain phases of development, autonomy and independence may be crucial objectives, but, in general, they are not considered overarching goals of behavior change.

### 3.3 Behavior change as a network of activity systems

**Figure 2:**
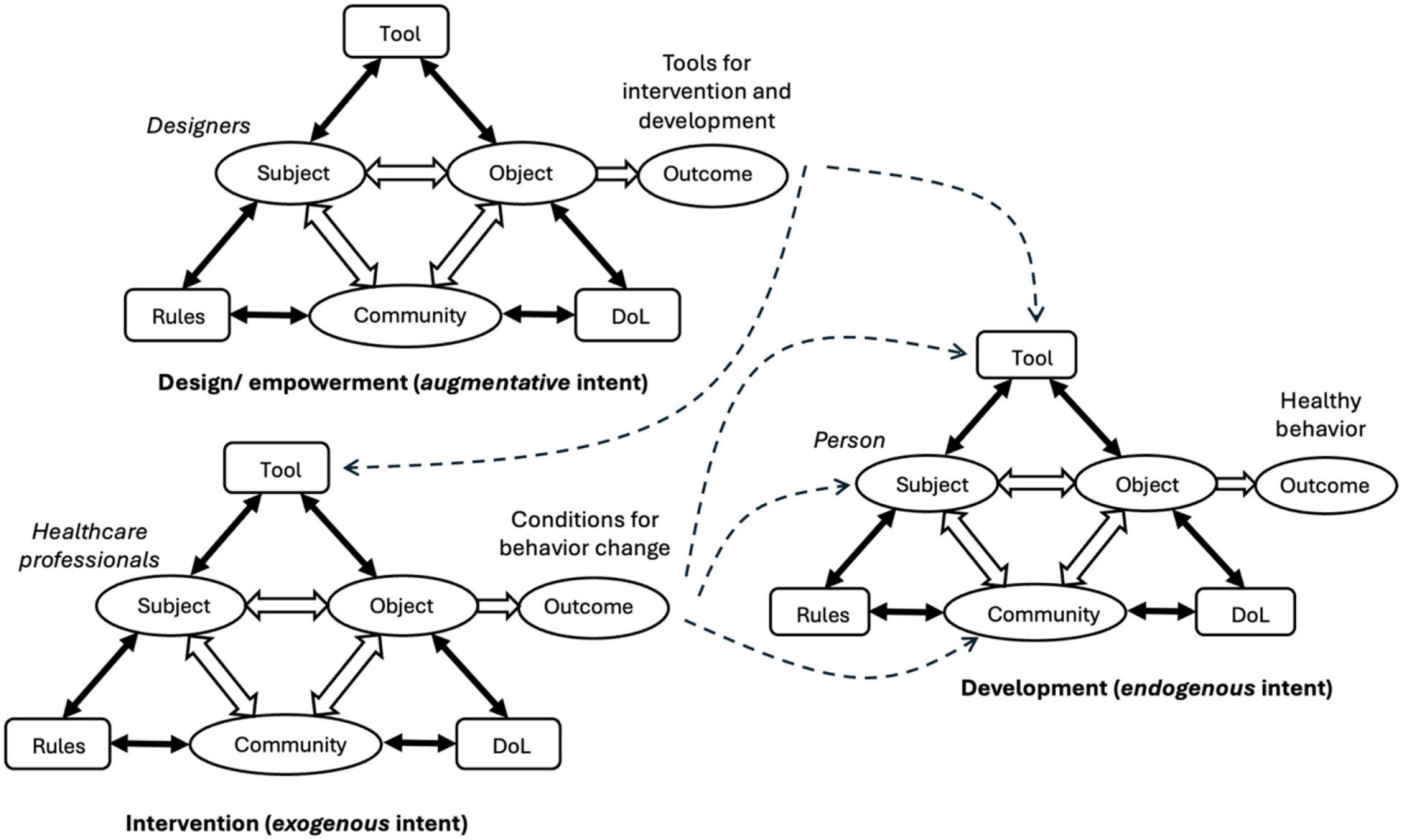
The proposed framework: behavior change as a network of three interacting activity systems (DoL, division of labor). Intervention system (exogenous) represents healthcare professionals creating conditions for change. Development (endogenous) represents the person's own transformation activities.

In this section, we introduce a framework of behavior change as jointly produced by three interacting activity systems: “intervention” (realizing an *exogenous* intent), “development” (realizing an *endogenous* intent) and “design/empowerment” (realizing an *augmentative* intent) (Figure 2). The desired outcome of the change, that is, a sustainable transition to a person's healthier behavior, is determined by the interplay between the systems.

Throughout this section, we refer to *exogenous behavior change* as change that is initiated and guided by healthcare professionals, and *endogenous behavior change* as the person's own contextualized efforts towards transformation. These categories generally correspond to, respectively, Fogg's terms “endogenous intent” and “autogenous intent” but ensure a closer fit with the focus of the present analysis. Moreover, interventions, which are often understood to be expert-led actions, in this framework are one node with a larger network of interrelated activity systems.

Table 1 summarizes the key terms used in this paper and how they relate to existing terminology in behavior change research.

**Table 1:** Key terminology used in the paper.

Term	Definition in this paper	Relation to existing concepts
Exogenous behavior change	Behavior change initiated and guided by external actors (e.g., healthcare professionals).	Loosely corresponds to Fogg's notion of “persuasive” intent.
Endogenous behavior change	Behavior change initiated and implemented by the person, reflecting their goals, and meaning-making in everyday life context.	Loosely corresponds to Fogg's notion of “autogenous” intent and is related to notions of intrinsic motivation and internalization in SDT.
Intervention activity system	Activity system of healthcare professionals who create conditions for person's behavior change.	Corresponds to professional healthcare practice and digital intervention systems.
Development activity system	Activity system of individuals who actively transform their own behavior.	Related to notions of self-regulation, internalization, and personal development.
Design/empowerment activity system	Activity system of technology designers producing artifacts that support both intervention and development.	Reflects the role of HCI and digital health design in shaping behavior change environments.

The *Subject* of the “intervention” activity system are healthcare professionals and its *Object* are a person's currently insufficient conditions for behavior change. The intended future, more-developed conditions are the *Outcome*. Various resources, such as behavior change techniques, are used by health professionals as *Tools* for creating the desired set of conditions. In the context of this paper, this activity system is understood to realize “exogenous behavior change” (which, as mentioned, approximately corresponds to Fogg's “persuasive intent”).

The “development” activity system is the activity system of the person who undergoes a behavior change. The person is the *Subject* transforming their behavior from the current state of being not sufficiently healthy (*Object*) to a desired state of a healthier behavior (*Outcome*). To achieve that, the person uses various *Tools* (e.g., interactive technologies). This activity system is understood as realizing “endogenous behavior change” (approximately corresponding to Fogg's “autogenous intent”).

Endogenous behavior change is a *contextualized* and *organic* transformation of a person's activities, characterized by a dynamic *re-distribution of agency between the person and health professionals*. It is contextualized, in the sense that behavior change should be analyzed in a larger context of activity transformation, which context affects, and is affected by, the behavior in question. For instance, switching to a more physically active lifestyle may cause wider developments of a person's activities, not limited to just being more physically active. A person may acquire new interests and social relations, while some of the existing ones may fade away or even disappear.

Furthermore, endogenous behavior change is organic: it is not just a straightforward implementation of a predefined step-by-step plan leading to a specified result, but rather a dynamically unfolding process of dealing with emerging issues (that is, resolving emerging contradictions), which requires a person's continuous attention and moment-to-moment decision making. The effectiveness of interventions and technological support, measured by their impact on this unfolding process, critically depends on how the therapeutic sessions and the use of technology are situated within this process as a whole.

Finally, to be sustainable, long-term behavior change should involve a redistribution of agency, and mutual transition of control, between the person and health professionals.. For instance, a person might initially need guidance and support from health professionals, eventually develop the ability to control of their behavior, and at some point, encounter an obstacle for further development, overcoming which could require an involvement of health professionals.

The “design/empowerment” activity system produces technological tools for the two types of agency “intervention” (exogenous behavior change) and “development” (endogenous behavior change). To do that, technology design should, on the one hand, identify and meet different requirements associated with these activity systems, and, on the other hand, support unfolding interactions and transitions between the activity systems. In this respect, the distinction between exogenous and endogenous behavior change, introduced in this paper, is different from Hicks et al.'s (11) distinction between intrinsic and extrinsic factors of behavior change. According to their distinction, intrinsic factors (e.g., having arthritis or a particular mindset) and extrinsic factors (e.g., living in a hot climate) are related to intervention—namely, they are to be taken into account to make an intervention personalized—rather than contrast different types of agency in behavior change.

### 3.4 The proposed framework and the intervention perspective: a comparison and boundary conditions

A comparison and boundary conditionsCompared to established models of behavior change such as the Fogg Behavior Model (17) and the COM-B system (18), the proposed framework operates at a different level of analysis. Rather than focusing primarily on the psychological determinants of individual actions (e.g., behavior as a function of motivation, capability, and triggers, or capability, opportunity, and motivation as core determinants of behavior) it conceptualizes behavior change as emerging from the interaction of multiple activity systems, including healthcare interventions, individuals' everyday activities, and technology design. For example, interventions targeting capability or motivation may be viewed as tools employed within the intervention activity system, while the individual's efforts to integrate new practices into everyday life correspond to transformations within the development activity system.

The proposed framework is intended to extend, rather than replace, intervention-oriented perspectives on behavior change. Instead of suggesting an alternative view of behavior change, it presents a potential extension, which is consistent with, and in some ways supportive of, the “intervention view”. In particular, the main areas of concern in relation to behavior change from an activity-theoretical perspective include motivation, goal setting, skill development, and social context management. It is easy to see that these areas generally correspond, for instance, to the elements of the COM-B model introduced by Michie et al. (18).

Intervention approaches remain essential, especially in clinical treatment programs and acute conditions requiring strict adherence, where healthcare professionals carry responsibility for treatment and patient safety. At the same time, long-term health management, lifestyle change, and relapse prevention often require individuals to integrate new practices into existing activities and social context. Arguably, because of its focus on contradictions, agency shifts over time, and support of mutual transitions between exogenous and endogenous change, the proposed framework can be particularly useful for analyzing and designing technological support these longer-term developmental processes.

## 4 Implications for the design of behavior change technologies

There is a complementarity and also contradictions between the exogenous and endogenous perspectives elicited through the lens of activity theory. Particular challenges in and implications for designing endogenous technologies (that is, technologies supporting endogenous behavior change) and exogenous technologies (that is, technologies supporting exogenous behavior change) are identified and discussed in the following sections.

### 4.1 Technologies for exogenous and endogenous behavior change

According to activity theory, technologies are mediating artifacts, which support people in achieving their meaningful goals. As shown in Figure 2, behavior change comprises three interrelated activity systems, and technologies that constitute the outcome of the activity system of design can mediate both health professionals' activities and the persons' own activities. On the one hand, a technology may be designed as a health professionals' proxy that constitutes a part of the intervention and thus supports exogenous behavior change. In that case, the technology can be employed to provide reminders, convey tasks and instructions, monitor progress, and in other ways serve as a virtual extension of the therapist. The technology, therefore, is essentially an extension of the “person-therapist” interaction beyond the time and space limits of face-to-face sessions. The authority structure of these virtual interactions should, arguably, be expected to be similar to that of face-to-face sessions: it is the health professionals who mostly decide, based on their experience and domain knowledge, what the desired outcome of the person's efforts should be, what indicators to monitor, what notifications to send, when to send them, etc.

On the other hand, a behavior change technology can be specifically designed to support endogenous behavior change activities. In that case, it is the person themselves who decides how and when to use the technology, and whether to use it at all, to support contextualization and self-control. Accordingly, a key purpose of this type of technology is helping the person to implement the intended behavior change in their particular life situation despite potential obstacles, both external and internal ones. An additional purpose of the technology is ensuring the sustainability of a behavior change. For that purpose, the technology should be designed to help the person maintain (and preferably develop further) the newly acquired healthy behavior when the intervention is over.

These two roles are not mutually exclusive and can potentially be combined within the same interactive artifact, e.g., a mobile app. However, the distinction is important, as it implies two different orientations in the design and use of behavior change technologies.

To be sure, there are apparent similarities between technologies supporting exogenous and endogenous behavior change activities. Not only is the transition to a healthier behavior their common overall objective, their specific goals also significantly overlap. They both include, for instance, keeping the person motivated, facilitating learning and skill development, optimizing planning, and managing social relations to promote the desired behavior change, which goals are traditionally associated with achieving a successful behavior change (18). At the same time, the design of exogenous and endogenous technologies is associated with somewhat different priorities, sensitivities, and assessment criteria—which are collectively referred to here as “design orientations” (see Table 2).

**Table 2:** A comparison of technologies for exogenous vs. endogenous behavior change activities.

Behavior	Behavior change activities	
	Exogenous design orientation	Endogenous design orientation
Authority (who is in charge)	Health professionals, other experts	Person
Responsibility (for accuracy and adhering to regulations)	Intervention-providing organization, enacted by health professionals	Technology-providing organization, person
Setting	Public (physical or virtual)	Both public and private
Support of	Setting up conditions	Carrying out change
Main challenge	Implementing target behavior	Resolving emerging contradictions
Scope of transformation	Specific behavior	Person's life
Person’s privacy	Lower	Higher
Interaction style	Can be formal	Can be playful

The most obvious difference between exogenous and endogenous technologies, reflected in the terms themselves, is in the *authority*—respectively, of health professionals or persons themselves—over the type of interaction supported by technology. The difference has implications for adjusting functionality and setting rights and permissions for concrete digital artifacts (i.e., defining the “owner” of the technology in question).

Related to authority is *responsibility*. The intervention-providing organization has to comply with regulations that assert who is accountable in case the technology causes harm or is faulty in some way^4^. As a consequence, it has to be clear when the responsibility begins and ends (i.e., the intervention) and what the scope of responsibility is (i.e., specific behavior or behaviors). For endogenous technology, the responsibility depends on which company or healthcare organization providing the artefact. However, it is commonly viewed that the person themselves is responsible for the use of digital technology and whose intent is mediated, also in the case of commercialized behavior change technology with a clear intent to sell person-tailored content and advertisements. In the following, we assume that there is an ideal case of endogenous technology without embedded intents from a third party.

While exogenous and endogenous technologies have the same overall objective, the aspects of behavior change that they *aim to support* are somewhat different. Exogenous technologies are used for establishing appropriate conditions for, as well as planning and assessment of, a behavior change. Endogenous technologies, on the other hand, are intended to support the person in actually carrying out the change. The latter is not a direct and straightforward outcome of exogenous behavior change activities. Instead, it is a situated action (61), which dynamically emerges in the given context rather than simply implementing a predefined plan.

Different types of support provided by exogenous and endogenous technologies mean that the *main challenges* that the technologies help to address are also different. The intended outcomes of using exogenous technologies are defining the conditions for behavior change and managing the transition from the current set of conditions to the desired set of conditions. In case of endogenous technologies, the main challenge may be not how to define the outcome (the target behavior may be relatively obvious) but rather how to navigate the situation at hand and resolve emerging contradictions between the goals of behavior change and potential conflicting factors (including the person's own resistance to the change).

Another difference concerns the *scope of the transformations*, which are supported by the technologies. The application scope of exogenous technologies is clearly defined and limited to certain types of behavior and conditions for the emergence of these types of behavior. The scope of transformation supported by endogenous technologies cannot be defined in a similar way; it depends on how much the change transforms the person's life as a whole. In some cases, the transformation can be local, while in other cases a person may need to make major adjustments in their environment and daily routines to make the change possible. A local change may also naturally lead to other, unanticipated changes, either positive or negative. Therefore, the application scope of endogenous technologies can only be defined by the person themselves, depending on their actual context. Crucial in this context is the sustainability of technology, considering that the scope of transformation, i.e., development, is ongoing across the lifespan.

In addition, exogenous and endogenous technologies may differ in the level of the *person's privacy*, which may be lower when technology use is a part of an intervention controlled by health professionals, and *interaction style*, which can be more formal in case of exogenous technologies to reflect the reliability and accountability of the healthcare provider, and more playful in case of endogenous ones.

These design orientations, summarized in Table 2, elicit fundamental challenges for design of behavior change technology aimed to serve both exogenous and endogenous purposes.

### 4.2 Intelligent agents: digital companions vs. “virtual therapists”

To be effective and efficient, technologies for behavior change do not have to be intelligent. Such tools as scheduled reminders, To Do lists, or instructional videos can be quite helpful despite being simple. At the same time, advancements in AI including generative models, capabilities for advanced processing, learning, and communication open promising new ways of for technologies to promote healthy behavior and, in particular, leverage the potential of *social* human-agent interaction.

Research has found that people naturally produce social responses to interactive technologies (62), and especially intelligent agents (63, 64). Technologies, which are deliberately implemented as “social actors”, have additional advantages in the context of behavioral change. Serving as social actors that have a persistent presence in a person's everyday life, they can capitalize on the person's social responses to positively impact motivation, learning, and control.

In general, intelligent agents can be implemented to support either exogenous or endogenous behavior change. In the former case, an agent may take on the social role of, e.g., a coach or an expert, which are exogenous in character, and use its communication capabilities to answer the person's inquiries and initiate interactions as a part of an intervention. In the latter case, an agent serves as an assistant, alter ego or companion, which roles are endogenous in their purpose taking as outset the person's viewpoint (65, 66).

#### 4.2.1 Virtual therapists

Intelligent agents designed to support exogenous behavior change typically take the form of virtual therapists, coaches, or clinical experts (38, 40). They usually mirror approaches of professional healthcare and often follow protocols, checklists, motivational interviewing, or behavior change models such as COM-B. The agent drives the interactions, gives recommendations, and evaluates compliance based on predefined metrics. These agents may deliver structured behavior change interventions, ask progress-based questions, provide motivational encouragement through expert guidelines, and adapt the feedback given based on data collected.

While these agents are mediums for continuity and accessibility for healthcare professionals beyond face-to-face sessions, they are still mirrors of traditional interventions: technology as a means of implementing healthcare remotely.

#### 4.2.2 Digital companions

In contrast with exogenous agents, intelligent agents designed for *endogenous behavior change* can better be described as digital companions. These agents can take the form of assistants, alter egos, or empathic confidants that operate with the user's own motives, context, and goals. Rather than delivering externally imposed plans, endogenous agents co-construct behavior change strategies through dialogue, reflection, deliberation, and dynamic adaptive support (67).

Digital companions may elicit personal values and long-term goals, reflect back contradictions between goals, offer context-sensitive suggestions as opposed to external commands, allow for emotional expression, empathy and non-linear interactions, and support users in overcoming barriers and setbacks while reinterpreting them in a constructive manner.

Designing digital companions requires but is not limited to building sustainable rapport with the user, privacy sensitivity, and working with ambiguous data as a person's emotional state can vary from one day to another. Digital companions also need to be adaptable in relation to the user, changing their role and tone depending on the user's mood, goals, or developmental phase. In this way, endogenous agents are more personalized and dynamic, also allowing the user to have a say in the construction of their own behavior change intervention rather than be bound by external expert guidelines (68).

#### 4.2.3 Hybrid design and implications

It is important to note that exogenous and endogenous agents are not mutually exclusive. Successful designs may implement both or allow the user to toggle between them. For instance, an agent might begin as a healthcare expert following clinical guidelines and gradually transition into a more empathic companion as the user builds up confidence and independence. This flow can be designed explicitly, supporting a transition from assisted to self-guided change.

Such role-switching mechanisms are described by Kilic et al. (65) by allowing the agent to adopt different roles such as healthcare expert, alter ego, or empathic companion depending on the user's preferences in the change process.

The distinction between exogenous and endogenous agents also highlights important implications for trust, accountability, and ethics. Users may be more likely to disclose sensitive information to companion-like agents, requiring robust privacy safeguards. Furthermore, overly authoritative agents may be effective in the short-term but raise risks of loss of engagement if they don't resonate with users’ own approaches and experiences. So, long-term engagement may be dependent on the agent's ability to adapt and evolve with the user, not just react to static models and protocols. Therefore, intelligent agents must be designed with flexibility in role as well as transparency in intention to ensure their presence empowers rather than undermines human agency.

#### 4.2.4 Ethical considerations for intelligent agents

It is important to address the ethical and safety questions alongside the design concepts outlined in this paper, especially when integrating LLMs into digital health contexts. Furthermore, an ethical distinction must be made between AI systems functioning as clinical advice tools vs. reflective digital companions. The former, corresponding to the exogenous orientation mentioned in Section 4.2.1, operates within defined protocols and has clinical accountability. Their recommendations may have a direct effect on the person's health and therefore should be held to the regulatory standards applicable to clinical decision support (e.g., EU legislation on medical devices and the EU AI Act). In comparison, digital companions, corresponding to the endogenous orientation, may not be designed to give advice but to support self-reflection, motivation, and empower the user aligning with the notion of wellness apps. Such systems do not aim to replace clinical support but augment the user's autonomy and development. However, due to the limitations of current GenAI technology, applications built on LLMs will also generate advice which may not be grounded in medical expertise nor aligned with what an individual needs in a situation, which risks reinforcing ill-health (69).

Due to such concerns, some safety measures must be considered when designing these systems. Digital companions that engage with users in sustained and personal dialogues might accrue sensitive data about the person. This warrants strong data protection, including data ownership, storage, access, and the conditions under which it may be shared with healthcare providers or researchers. In addition, the system must be transparent, i.e., users should be clearly informed that they are interacting with an AI system, not a healthcare provider or peer. The system's limitations as well as capabilities must be clearly communicated in accessible language to the users. Moreover, in endogenous spaces, transparency can help users understand what data is being used in personalizing the companion's responses and in what way.

Furthermore, hybrid systems that combine the two orientations can introduce accountability concerns. When a person acts on content spanning both spaces, it may not be always clear whether the recommendation originated from clinical guidelines or AI shaped by the user's dialogue history. Each module must be clearly identifiable as AI generated or expert recommended. In the end, the system provider will need to assure the adherence to regulations, e.g., CE-marking if needed, and be held accountable for all content of the system.

Finally, it should be noted that ethical considerations are not limited to the above only. The framework we present in this paper recognizes the persons as agents of their own development and not only mere objects of intervention. Keeping this in mind when designing AI companions means actively designing *against* manipulation and user autonomy erosion, which are risks that must be accounted for in the context of generative AI technologies.

## 5 Selected design concepts and issues

Based on the activity-theoretical framework outlined in this paper, we identify a set of interrelated design concepts that can serve as a guide for the development of technologies for behavior change particularly those aimed at supporting endogenous (self-driven) transformation. These concepts reflect key aspects of activity theory, including distributed agency, contradictions, and the role of artifacts in mediating subject-object relations. In the following, we elaborate on five critical issues that emerge from this perspective and discuss how they inform the design of behavior change technologies. The critical issues and concepts are further discussed through examples in Section 6 where we give examples from research on intelligent digital companions.

### 5.1 Exogenous and endogenous interaction spaces

There is a basic *distinction between exogenous and endogenous technologies*. Different types of technologies can be implemented as separate artifacts (e.g., different mobile apps), but other options are also possible. For instance, these types can represent different interaction spaces within the same artifacts, namely, a space shared with healthcare professionals and a space only reserved for the person themselves. Yet another possibility is to have a shared and private version of individual functions. The separation allows for both structured guidance and autonomy at the same time. This distinction also allows the person to situate themselves within a broader network of activity systems while having a say over their behavior change.

### 5.2 Contradiction as a driver for development

A key focus in the design of endogenous technologies should be on the *contradictions* that drive development. It can be achieved through explicating contradictions in a person's behavior change activities, embedding tools for reflecting on multiple, potentially competing goals and commitments, and prioritizing the goals and commitments when making specific behavioral choices. Behavior change technologies that utilize this framework can be designed to help individuals realize this. For example, when a suggested activity (e.g., go running to increase physical activity) clashes with other commitments (e.g., taking care of kids after work), the system might elicit the user's reasoning, help explore alternative paths, or help re-frame the behavior in relation to broader personal values and commitments. Such a design aids individuals' development not by making the activity simpler or discarding it but helping users engage and uncover these contradictions.

### 5.3 Supporting holistic and sustainable transformation

Endogenous technologies should support a *holistic* activity transformation, i.e., they should be designed not only to structure individual activities but to help the evolving activity system of the user. For instance, adding functionality to visualize progress to monitor change, while also providing means to define and revise long-term objectives as well as short-term goals rather than focusing on low-level, fragmented changes could evoke persistence of a change. The behavior change technology could also display envisioned consequences of a change as anticipated desirable futures, in terms of improved health, improved balance between recovery and other activities serving different motives important to the person. Supporting a holistic activity transformation entails recognizing behavior change as something embedded in daily life, not simply within a window of intervention (22). Instead of tracking adherence to interventions, behavior change technologies should support people in their desire to reshape their actions, thoughts, and personal motives.

### 5.4 Enabling appropriation

A key issue from the point of view of the proposed framework is *appropriation*, or how users make technology “their own” and use it for their own purposes. Appropriation, in this sense, does not only refer to the customization of an app's interface, but also to the redefinition of its use: the way people integrate it into their own daily life. For instance, when monitoring their progress, a person may include additional indicators, not shared with the therapist, for their private use. In general, appropriating functions such as adding personal, potentially intimate, aspects of a user's life, not intended to be shared with others, can prompt positive attitude towards the technology and form a richer basis for tailoring the support provided by the technology to a person's needs in way that is more useful and meaningful to them. This may include eliciting motives that are not within clinical guidelines, tracking progress using personally relevant data, or recording reflections that are only intended for the user to see. Doing this gives the user the power to reshape the tool for personal development but also gives users autonomy.

### 5.5 Supporting transitions in agency

Explicitly structuring and assessing a transition between assisted and unassisted behavior change is too a way to achieve a successful, sustainable behavior change. In some cases, a person may begin their behavior change journey under the watchful eye of a professional and then internalize and sustain the newly acquired behavior. In other cases, the person may turn for help (or offer help to others) if acting independently does not bring the desired results. By facilitating such agency shifts, behavior change technologies can help individuals move from external guidance to appropriation and back, that is, support a *dynamic individual/social re-distribution* of activities through a coordinated use of both exogenous and endogenous technologies.

## 6 The design orientations exemplified

The distinctions outlined above between exogenous, endogenous and augmentative perspectives are not only theoretical but are directly applicable to real-world design of eHealth tools for behavior change. In this section, we provide examples, each reflecting a particular design orientation in terms of agency, responsibility, and design goals, including one from the authors' own work. A comparison of the orientations is outlined in Table 2. These examples not only illustrate how the design orientations can be implemented in real-world technologies but also help clarify the tensions and opportunities that can emerge when developing eHealth systems. In particular, they underline how technology can shift from being a proxy for external instruction to a tool for internal transformation, or ideally, a bridge between the two.

### 6.1 Exogenous technology: diabetes management app

A typical example of an exogenous system is a diabetes management app integrated with electronic health record systems in clinical settings such as Glooko. These apps are prescribed by healthcare providers and are used for sending reminders to patients to take insulin or medications as well as log blood sugar levels according to defined schedules. They also send automated alerts to clinicians if the patient doesn't adhere to the schedule and provide static educational content to the patients that are in line with professional guidelines.

The app's function mirrors the intent of the healthcare professionals who determine the behavior change and how change should be monitored. This establishes the patient's role as a compliant user whereas the app serves as a proxy for the clinician. The app is a good example of a low-autonomy design where adherence tracking, accurate data, and compliance are the main concerns.

### 6.2 Endogenous technology: generative chatbots

As contrast to the exogenous example, the commonly used generative LLMs such as ChatGPT can be used by anyone to explore emotions, habits, and daily reflections (70). Other well-known examples include Replika or Nomi. Unlike clinician-prescribed technologies, it encourages self-expression without predefined targets, responds to expressions, and supports long-term engagement by evolving based on how the user chooses to interact.

In a traditional sense, there is no intervention. Instead, the user takes control of how the technology is used, what it tracks, and what goals are pursued. This is in line with the endogenous orientation, where behavior change is implicit in the user's self-reflection and daily life. It is, in a way, a mirror for self-development and not a prescription. The main expected benefits with such technologies are flexibility, user agency, and rapid access to general and personally relevant information. The main concerns are the limited trustworthiness of the information, potential triggering and enhancement of negative emotions, and privacy.

### 6.3 Hybrid technology: behavior change support app

As a case study for demonstrating the design orientations elicited in Section 4, we introduce the mobile application StarCoach, presented in Lindgren and Kilic (71). StarCoach is an example of how the two above orientations can be integrated in a single system with clear accountability boundaries, referring to the authority responsible for the health-related content, while the overall authority and responsibility lie with the user (Table 2). While the app is designed to support setting up conditions for behavior change (exogenous), the primary support is focused on carrying out the change resolving emerging contradictions (endogenous). The healthcare perspective elicits specific behavior, while the person can place their goals for behavior change within their daily living over time. The person's privacy is high, and interaction style can be playful if the person shapes the content such (Table 2).

**Figure 3:**
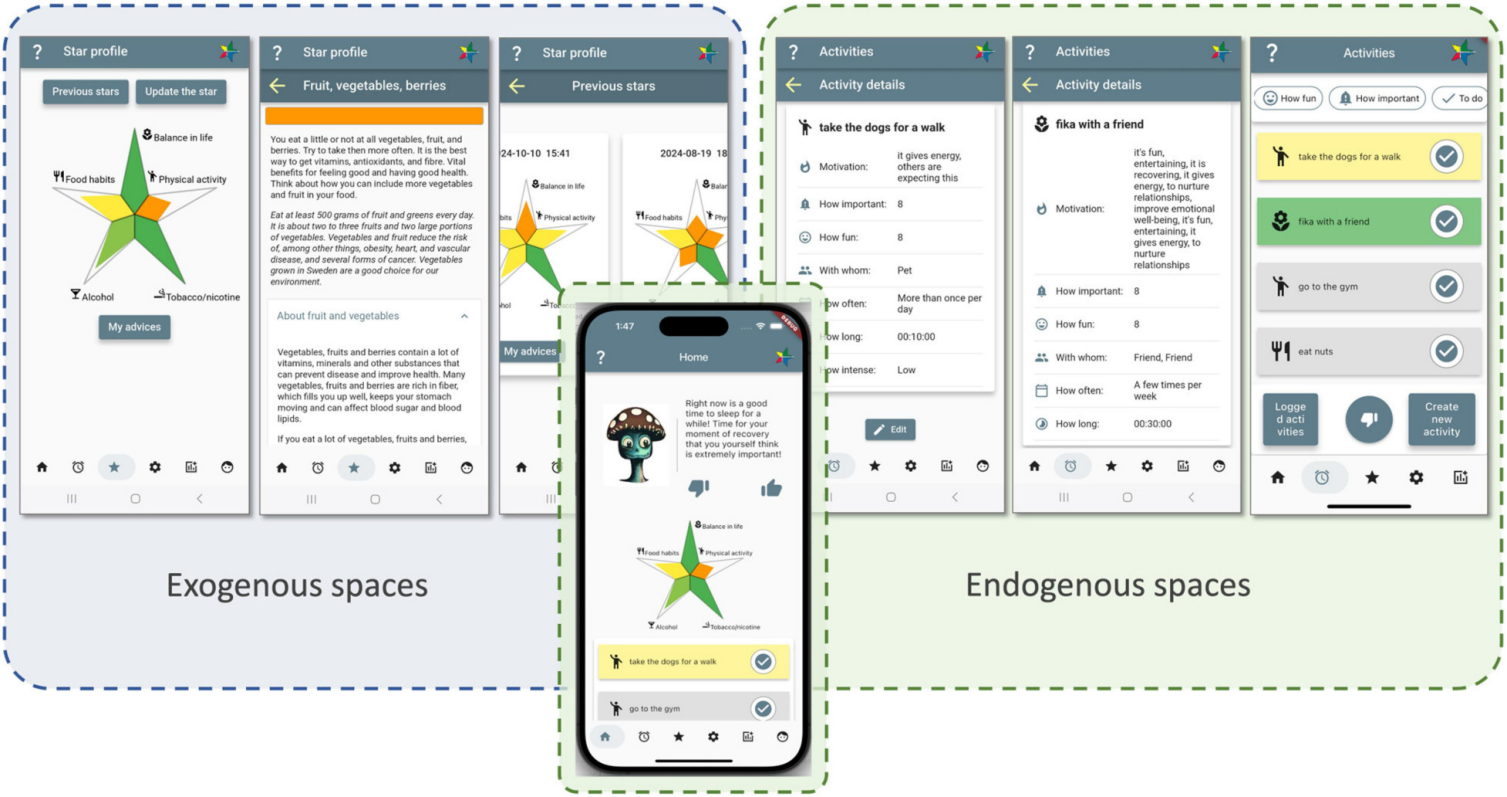
Starcoach as a hybrid behavior change system: exogenous and endogenous spaces in a single application. The exogenous space embeds evidence-based advice written by domain experts. The endogenous space supports user defined short-term goals and self-reflection.

StarCoach includes an exogenous space, an endogenous space, and a digital coach that shifts between roles ranging from healthcare expert to alter ego, depending on user preferences (Figure 3). The agent also engages in dialogue, mediating between clinical recommendations and personal realities. The strength of the app lies in how it supports endogenous transformations: the person is not only following clinical advice but actively integrating it into their own activity systems, resolving contradictions, negotiating priorities, and personalizing behavior change.

#### 6.3.1 Exogenous and endogenous spaces in StarCoach

On the exogenous side, StarCoach embeds a number of BCTs promoting the exogenous perspective, including long-term goal setting, problem solving, self-monitoring and credible source (71). Other BCTs could be viewed as primarily supporting the endogenous perspective such as eliciting pros and cons, incompatible beliefs, and feedback on behavior. In this particular example, the main modules embedding the BCTs can be viewed as either exogenous or endogenous. Furthermore, they are also, to a different extent, relevant for supporting development and managing contradictions. Therefore, first, the distinction is made between exogenous and endogenous spaces and functionalities: (exogenous) (i) a separate page for information and tasks related to healthcare, (ii) another page for the long-term goals defined by healthcare, and related person-tailored advice composed by healthcare representatives; (endogenous) (iii) a page for own short-term goal-oriented activities, defined freely in terms of what to do, what motives they serve, and how often to do the activity; and (iv) a digital coach, adopting different roles and positions according to the user's preferences, and which captures barriers to adherence, representing contradictions (Figure 3). While initial evaluation studies show how participants elaborate on their goals and activities across the spaces, long-term effects of using the different spaces and the management of contradictions in terms of barriers need to be studied in future studies.

#### 6.3.2 Contradiction as a driver for development

To elicit contradictions, the user can specify motives that may be contradictory, and the digital coach initiates dialogues to elicit barriers when an activity suggested by the coach is negatively received. The user can revisit their reasons for not adhering to the coach's suggestions. For example, if a user rejects a proposed activity, the digital coach initiates a dialogue to explore the reasons which can unveil potential conflicts between health goals and corresponding life demands (e.g., “No time due to taking care of kids after school”.).

Through structuring these contradictions as opportunities for self-reflection, this allows moving beyond persuasion and leads to awareness of tensions within daily activity systems. This supports situated decision-making rather than externally enforced compliance, augmenting the user's role as a developing subject as opposed to an object of change.

#### 6.3.3 Supporting holistic and sustainable transformations

Unlike many health apps that focus on limited behavioral metrics such as a step counter or a calorie tracker, StarCoach supports a long-term holistic perspective on behavior through addressing multiple, inter-related lifestyles, on a short-term, as well as long-term basis. The long-term vision is represented as a star-shaped palette of optimal lifestyles represented by green color; the path forward may lead through colors representing different levels of achievements for the different lifestyle domains.

By giving the users the opportunity to connect daily actions with evolving dynamic long-term goals, StarCoach supports Leontiev's and Engeström's multi-level activity structure through linking operations (e.g., going for a run), actions (e.g., building stamina), and overarching activities (e.g., being more present for family).

#### 6.3.4 Enabling appropriation

To support appropriation, the user can add content that is not complying with the healthcare organization's intent and purpose with the activity functionality, also as response to the digital coach's request for reasons and barriers, which the user can use as their personal notes. The question of what information is stored where and for which purposes, is still open, since data is currently stored for research purposes only. However, the general view among healthcare experts was that it should be the person's own property (72). The data can be viewed as personally mediated artifacts, in terms of Activity Theory and expand upon the internalization of the behavior change while respecting the boundaries of privacy.

#### 6.3.5 Structuring transition from assisted to autonomous change

The support for transition from assisted to unassisted behavior change activities starts when the person leaves the health–check-up and motivational interviewing session with a nurse. The content from the motivational interviewing session is carried over into StarCoach, embedding the intervention into the user's own personal device to manage the transition. The transition can be supported by the digital agent present in the app through dialogues eliciting barriers but also delivering tailored advice (65). This approach allows the user to revise, discard, or redefine health goals post-intervention, empowering them by giving ownership and adaptability to be the primary decision maker in the transition. In this way, the exemplified application not only extends the outreach of traditional interventions but also sets into motion the shift from exogenous to endogenous activity systems as a process of redistribution of agency.

#### 6.3.6 Summarizing design implications of the proposed framework

The example elicits the difference between exogenous and endogenous behavior change regarding main challenge and scope: while the person resolves contradictions relating to everyday life, in their pursuit of improved health, healthcare focuses on the lifestyle change that they have resources and authorization to support (Table 3). The authority is manifested in the content of the person's version of the application: healthcare representatives are responsible for the module for long-term lifestyle goals and related advice, partly channeled through the digital coach, but cannot control what the person chooses to prioritize in terms of short-term goals, their motives, and topics of the dialogue with the digital coach. The person, on the other hand, is not authorized to change the content of the recommendations provided by the module for long-term goals and related advice authored by healthcare but is in control of the activity and digital coach modules. As a consequence, the responsibility to manifest change stays with the person, while healthcare is responsible for the evidence-based and best practice-based recommendations. This separation reinforces distinct modes of agency; the long-term lifestyle goal module anchoring healthcare's contributions, while the activity module and agent are purposed to promote ownership, reflection, and sustainable engagement beyond the intervention window.

**Table 3:** Comparison of three technology examples across orientations illustrating how they can be implemented in real-world technologies.

Example	Orientation	Who sets the Goals?	Tech role	Interaction style	Primary context
Clinician-guided diabetes app	Exogenous	Health Professional	Professional Proxy	Structured, Serious	Clinical and Home Care
Journaling app	Endogenous	The user	Reflective Tool	Informal, expressive	Daily life
StarCoach	Hybrid	Shared: healthcare & user	Mediating companion	Mixed: serious/empathic	Transition from care to autonomy

StarCoach also exemplifies how activity theory can guide the design of such interactive systems respecting the user agency while still being connected to professional expertise. By utilizing principles such as contradiction and distributed agency, the app supports individuals in becoming agents of their own behavior transformations in a more developmental way rather than through external pressure or persuasion.

## 7 Concluding discussion

In healthcare practice, behavior change is commonly considered as predominantly an outcome of an intervention performed by health professionals. Accordingly, the objective of behavior changes is viewed as employing advanced models and techniques to recognize and correct a deficiency in a person's conditions for adopting a healthy behavior [e.g., (18)]. While the intervention perspective is supported by solid empirical evidence and proved to be practically useful, it tends to downplay the role of a person's own agency. In our view, it is a significant limitation, as, arguably, a precondition for an effective, ethical, and sustainable change is recognizing the person not as a target of change, but as its architect.

Addressing this limitation, human-technology interaction research has been increasingly exploring ways to provide technological support for the agency and autonomy of persons as subjects transforming their own behavior [e.g. (20, 21, 23, 55, 57)]. This paper contributes to this research by proposing a framework, informed by activity theory, which depicts technology-supported behavior change as an interplay between activities of technology designers, health professionals, and, most importantly, the persons themselves.

According to Bennett et al. (42), a key distinction characterizing current HCI research on agency and autonomy is “independence vs. interdependence”, that is, emphasizing either an independence of external influences (and often referring to *autonomy*) or the integration of a human agency into the social context. This distinction also applies to human-technology research on behavior change. Some studies, especially those informed by SDT, foregrounding achieving autonomy/independence, and other ones foregrounding holistic, socially embedded transformations of human activities and lived experience. We also argue that the latter, while providing valuable general insights, need to be further elaborated to serve as a more concrete foundation for the design of behavior change technologies. The activity theoretical framework, presented in this paper, and providing an account of a person's agency as a part of a network also comprising agencies of other subjects, is intended as a step toward this goal. In particular, while foregrounding endogenous behavior change activities, the proposed framework does not mean to downplay the role of exogenous ones. Instead, it points out that both are important, and their integration is key to a successful behavior change. It should be noted that, in our view, there are no irreconcilable differences between the analyses of agency and autonomy in behavior change, foregrounding dependence or interdependence. While they may differ in their *relative* emphases, they represent complementary and largely overlapping perspectives on how technologies should be designed and used to support people in transforming their own behavior. Arguably, behavior change research requires combining these perspectives—which the framework we propose is intended to facilitate.

Importantly, the relationship between professional intervention and personal development should not be understood as a one-directional process in which externally guided interventions are gradually internalized by individuals. Instead, behavior change often involves ongoing interaction between these forms of support. Individuals may reinterpret or adapt professional recommendations within their everyday activities, while also returning to professional guidance when new challenges arise. From this perspective, behavior change support can be understood as a dialogic process, involving continuing negotiation between externally guided intervention and self-directed development.

The conceptualization of behavior change in the proposed framework has both theoretical and practical implications. Theoretically, it invites behavior change researchers to consider the situated, socially mediated, and temporally unfolding nature of behavior change while also highlighting the role of contradictions as drivers of development. The framework complements existing theoretical accounts, which mostly focus on motivation or persuasion. Such focus, while important, is not sufficient, as a person may be fully motivated and convinced that a change is needed but still struggle to fit it with their other demands and constraints. In such cases additional motivation and persuasion efforts may be counterproductive, reinforcing a sense of failure or lack of sense of agency.

The framework also emphasizes the need for technologies that support individuals in navigating through complex and often conflicting goals in daily life. It also highlights a set of design issues related to the coordination of technologies built to expand the reach of professional interventions and those that empower user-driven transformation. Addressing these issues can inform decisions around system design (e.g., spaces), interaction style (e.g., coaching vs. empathy), personalization, and ethical responsibility. Furthermore, by addressing these issues, AI-based personal health technology can be created that not only support compliance but also support behavioral appropriation, autonomy, and sustainability.

The exemplified behavior change application illustrates how the outlined principles can be used in the real world. The app is a proof of concept for a behavior change support technology that integrates exogenous and endogenous functions, supports contradiction resolution, appropriation, and enables transformations from assisted to self-driven change. In doing so, it shows how activity theory can serve not just as an analytical tool, but also as a guide for designing intelligent, human-centered health technologies.

The proposed framework contributes to current research emphasizing the importance of explicitly recognizing and supporting persons as agents of behavior change. We argue for a coordinated use of employing exogenous technologies, which deliver health professionals' instructions or encouragements, i.e., challenge people, and endogenous technologies, which help people deal with the challenges. Such technologies should support behavior change as a transformation of a person's entire life situation and provide tools and spaces for accomplishing the transformation. We also argue that a key role of intelligent agents is serving as “understanding” and supportive companions, who are always on the person's side, complying with ethical and responsible AI guidelines.

Yet to be developed are evaluation criteria to assess to what extent behavior change applications are supporting endogenous and exogenous activities respectively, and how they respectively and together support behavior change goals in individuals.

## Footnotes

^1^Here the term “technique” does not refer to technology.

^2^The discussion here specifically focuses on health professionals. Behavior change activities initiated by other actors, such as general lifestyle coaches or educators, are beyond the scope of the present analysis.

^3^In this paper, “persons” is used to refer to the individuals whose behavior is being changed.

^4^The European Union AI Act: https://artificialintelligenceact.eu/
